# Development and validation of a hypoglycemia risk prediction model for hospitalized type 2 diabetes patients following laparoscopic colorectal cancer resection: a retrospective cohort study

**DOI:** 10.3389/fendo.2026.1932701

**Published:** 2026-08-28

**Authors:** Yeye Zhuo, Yundan Zheng, Jing Xu, Xinxin Li, Weiyi Huang, Leiquan Ji, De Cai, Yanrong Chen

**Affiliations:** 1 Department of Clinical Pharmacy, The First Affiliated Hospital of Shantou University Medical College, Shantou, China; 2 Department of Pharmacology, Shantou University Medical College, Shantou, China; 3 Department of Gastrointestinal Surgery Department, The First Affiliated Hospital of Shantou University Medical College, Shantou, China; 4 Department of Endocrinology, The First Affiliated Hospital of Shantou University Medical College, Shantou, China; 5 Department of Clinical Laboratory, The First Affiliated Hospital of Shantou University Medical College, Shantou, China

**Keywords:** laparoscopic colorectal cancer resection, nomogram, postoperative hypoglycemia, risk prediction model, type 2 diabetes mellitus

## Abstract

**Objective:**

To identify factors associated with postoperative hypoglycemia among inpatients with type 2 diabetes mellitus (T2DM) receiving laparoscopic colorectal cancer (CRC) resection, and to construct a predictive model for assessing hypoglycemia risk.

**Methods:**

This retrospective cohort study collected data on hospitalized T2DM patients who underwent laparoscopic CRC resection between January 2021 and June 2023, and randomized them into a training and an internal validation cohorts in a 7:3 ratio. Multivariate logistic regression was used to build a prediction model for in-hospital hypoglycemia after laparoscopic CRC resection and identify independent predictors for level 1 and level 2 hypoglycemia.

**Results:**

Among 720 hospitalized T2DM patients receiving laparoscopic CRC resection, the overall incidence of postoperative hypoglycemia was 16.7% (120 patients), including 98 level 1 cases and 22 level 2 cases. A multivariable predictive model was developed using five independent predictors: long T2DM duration, low preoperative day-1 fasting plasma glucose (FPG), American Society of Anes- thesiologists physical status grade > II, prolonged postoperative fasting, and prior long-term insulin therapy. The model yielded an area under the receiver operating characteristic curve (AUC) of 0.817 (95% confidence interval [CI], 0.774-0.859) in the training cohort and 0.751 (95% CI, 0.669-0.833) in the validation cohort for hypoglycemia prediction. Level 1 postoperative hypoglycemia was independently predicted by long T2DM duration, low preoperative day-1 FPG, and prolonged postoperative fasting; while level 2 hypoglycemia was associated with low body mass index (BMI), low pre- operative day-1 FPG, and elevated neutrophil (NEUT) proportion on postoperative day 3.

**Conclusions:**

The nomogram prediction model constructed in this study provided reliable risk estimation for postoperative hypoglycemia in hospitalized patients with T2DM undergoing laparoscopic CRC resection, and might serve as a practical adjunct to inform individualized clinical decisions and optimize perioperative glycemic management.

## Introduction

Globally, colorectal cancer (CRC) is responsible for the third highest incidence of malignancies and the second leading cause of cancer mortality (1). CRC occupied the second place in cancer prevalence in China, with Chinese patients comprising approximately 30% of all CRC cases worldwide (2). On the other hand, epidemiological and biological evidence indicate that patients with diabetes face a higher risk of developing CRC. Metabolic disorders in diabetes may promote CRC oncogenesis and cell proliferation by triggering inflammation and oxidative stress-related changes in signaling pathways (3). Meta-analyses have revealed a correlation between type 2 diabetes mellitus (T2DM) and elevated CRC risk, with relative risks varying from 1.2 to 1.4 (4, 5).

Indeed, surgery remains the primary approach for CRC treatment, which, together with chemotherapy and radiotherapy, constitutes the current clinical standard (6). However, compared with non-diabetic patients, patients with T2DM experienced a 16% increase in the risk of overall complications after CRC surgery and a 1.17-fold greater risk of 30-day postoperative mortality (7, 8).

Findings from observational and experimental studies support that perioperative intensive glucose control positively influences postoperative morbidity, lowering the risk of adverse outcomes (9). However, notably, a tight control of blood glucose was associated with an elevated risk of potentially hypoglycemia (10). Hypoglycemia is one of the most common complications in patients with T2DM, with the incidence of postoperative hypoglycemia in patients undergoing elective surgery up to 24.78% (11). Postoperative hypoglycemia was found to be related to increased mortality in elective craniotomy and cardiac surgery (12, 13). As evidenced by the study of Goh SNS et al. (14), perioperative hypoglycemia conferred a 19-fold higher likelihood of developing Clavie-Dindo grade ≥ II postoperative surgical complications among T2DM patients undergoing CRC resection.

The use of laparoscopy in colorectal procedures offers significant benefits to the general patient population, including accelerating postoperative recovery, reducing complications, and smaller incisions with minimal blood loss (15). However, the reduced surgical stress response associated with this approach may, counterintuitively, result in lower blood glucose levels in affected patients (16). Additionally, several surgery-specific factors following CRC resection, most especially postoperative long-fasting and the common provision of enteral and parenteral nutritional support, may also contribute to the risk of hypoglycemic events (17).

Several studies have preliminarily investigated the risk factors for perioperative hypoglycemia in patients with T2DM, with study populations covering all elective surgical procedures or gastro- intestinal surgery cohorts (11, 18, 19). Their clinical applications was not confined to a single category of surgical intervention. Variations in both surgical procedures and perioperative care across operation types may result in differing risk factors for perioperative hypoglycemia. As of yet, there is a paucity of published evidence focusing on the risk factors and prediction models for hypoglycemia following CRC surgery in individuals with T2DM. We conducted a single-center and retrospective cohort study to develop and validate a predictive model for postoperative hypoglycemia risk in hospitalized T2DM patients undergoing laparoscopic CRC resection. A secondary aim was to identify differential risk factors for level 1 and level 2 hypoglycemia, thereby supporting targeted preventive interventions.

## Materials and methods

### Study design and setting

A nomogram was constructed and validated in this investigation to forecast the risk of perioperative hypoglycemia among patients with T2DM following laparoscopic CRC resection through an observational retrospective cohort study. This study was conducted at a 1800-bed primary care hospital in China. Data were collected from hospitalized patients undergoing elective laparoscopic radical resection for CRC between January 2021 and June 2023. Ethical approval was obtained from the institutional review board of the First Affiliated Hospital of Shantou University Medical College (approval No. B-2021-088). All participant case information was collected anonymously. Informed consent was obtained from all individual participants included in the study.

Inclusion criteria included: aged ≥ 18 years; diagnosed with T2DM according to American Diabetes Association criteria (2020) (20), with a documented history of ≥ 3 months antihyperglycemic pharmacotherapy; perioperative glycemic control was maintained using glucose- lowering agents, including oral antidiabetic drugs, insulin, and incretin-based therapies; underwent elective laparoscopic radical CRC resection; routine point-of-care capillary (POCT) glucose (fasting, 2-h postprandial) was conducted during hospitalization, with at least ≥ 3 days of continuous pre- and postoperative monitoring.

Exclusion criteria included: type 1 diabetes mellitus, gestational diabetes mellitus, or other distinct forms of diabetes; emergency surgery, or ≥ 2 surgical procedures performed during the observation period; postoperative intensive care unit admission due to shock or other severe acute medical disorders; with a missing data rate above 40% across medical record variables (including clinical features, laboratory findings, and perioperative surgical details).

### Data collection and definition

Study variables were identified on account of previous literature (11, 18, 19, 21) and clinical experience. Pharmacotherapy-related factors referred to commonly used agents with documented hypoglycemic risk (22). All data were sourced from the hospital electronic medical records (EMRs) by two investigators who had undergone training. The study observation period encompassed the full hospital stay for the index surgery. To ensure data reliability, a review was conducted on 10% of cases, centering on key variables such as hypoglycemic events and medication documentation, alongside supplementary accuracy checks for all outliers and extreme cases. Data extraction covered the three dimensions outlined below.

1. Demographics and clinical characteristics: age, sex, body weight (BW), body mass index (BMI), T2DM duration, comorbidities (hypertension, hyperlipidemia, thyroid disease, and renal insufficiency), preoperative glucose metrics (glycated hemoglobin [HbA1c], preoperative day 1 fasting plasma glucose [FPG]; 2-hour postprandial plasma glucose [2hPPG], and 3-day preoperative mean plasma glucose [MPG]), routine biochemistry on admission (estimated glomerular filtration rate [eGFR], liver enzymes, and lipids), postoperative day 3 complete blood count (white blood cell [WBC] count, neutrophil [NEUT] proportion, and C-reactive protein [CRP]), and postoperative length of stay (PLOS).
2. Operative parameters: total operative duration, anesthesia delivery approach, American Society of Anesthesiologists (ASA) physical status grade (class II or higher), and duration of postoperative fasting.
3. Pharmacotherapy-related elements: long-term baseline medication exposure (preoperative continuation of pre-admission regimens), perioperative medication administration.

Blood glucose was routinely measured via POCT using standard-grade glucometers (Accu-Chek; Roche) during hospitalization. Postoperative hypoglycemia was defined as at least one bedside POCT blood glucose value < 3.9 mmol/L recorded during postoperative hospitalization (20). Furthermore, Level 1 hypoglycemia was defined as a measurable glucose concentration ≥ 3.0 mmol/L and < 3.9 mmol/L. Level 2 hypoglycemia was defined as a blood glucose concentration < 3.0 mmol/L, the threshold for neuroglycopenic symptom onset requiring immediate corrective action (20). According to the predefined grading criteria, cases with concurrent level 1 and level 2 postoperative hypoglycemia were assigned exclusively to the level 2 category.

### Sample size

Sample size was calculated in accordance with the established studies for risk prediction model development (23). The number of outcome events should be at least 5–10 times the number of independent variables incorporated into the multivariable model. At the study design stage, 10 candidate independent variables were predefined for inclusion in the final risk prediction model. A total of 89 hypoglycemic events were documented in the training cohort, meeting the prespecified sample size threshold. Based on the reported 24.78% incidence of postoperative hypoglycemia in patients with T2DM undergoing elective surgery (11), a sample size of at least 485 subjects was required for model development in this study.

### Statistical analysis

All data processing and statistical analyses were conducted in SPSS (version 26.0) and R (version 4.5.2). All *p* values were two-tailed, and *p* < 0.05 was considered statistically significant.

Missing values (BMI: 0.69%; HbA1c: 0.56%; CRP: 1.11%) were addressed with multiple imputation. To handle randomly missing data, multiple imputation with predictive mean matching was implemented using the R mice package, yielding five imputed datasets. The final dataset was derived by taking the mode across the five imputations, and a sensitivity analysis was performed to verify result robustness (**Supplementary Table 1**).

Patients were randomly allocated to the training and internal validation cohorts in a 7:3 ratio using the “create Data Partition” function in R, ensuring random distribution of outcome events between the two cohorts. The training cohort was used for variable screening and model construction, while the validation cohort served to validate the results generated from the training cohort.

The Kolmogorov-Smirnov test was used to verify normality of continuous variables. Normally distributed variables were presented as mean ± SD and between-group comparisons were performed with independent samples *t*-test; non-normal variables were expressed as median (Q1, Q3) and intergroup comparisons were analyzed with the Mann-Whitney *U* test. Categorical data (n [%]) were compared using the χ² test or Fisher’s exact test.

Pearson correlation coefficient analysis and variance inflation factor (VIF) test were applied to screen for multicollinearity across all predictors. Variables with significant correlations (*p* < 0.05) or elevated VIF values (> 10) were reviewed and refined to maintain model robustness and interpretability. Variables demonstrating collinearity were excluded, and all the remaining factors were entered into logistic regression to identify independent predictors of hypoglycemic events. Model discrimination was evaluated by the area under the curve (AUC) in the receiver operating characteristic (ROC) analysis, with values > 0.7 defined as good discrimination. Calibration ability was assessed using the Hosmer-Lemeshow test, calibration slope, calibration intercept, and Brier score. Decision curve analysis (DCA) was applied to quantify the model’s net clinical benefit.

To guard against overfitting and calibrate estimates of model performance optimism, 1000-iteration bootstrap resampling was implemented. A nomogram was further generated to enable more intuitive visualization of the results.

## Results

### Patient characteristics

**Figure 1:**
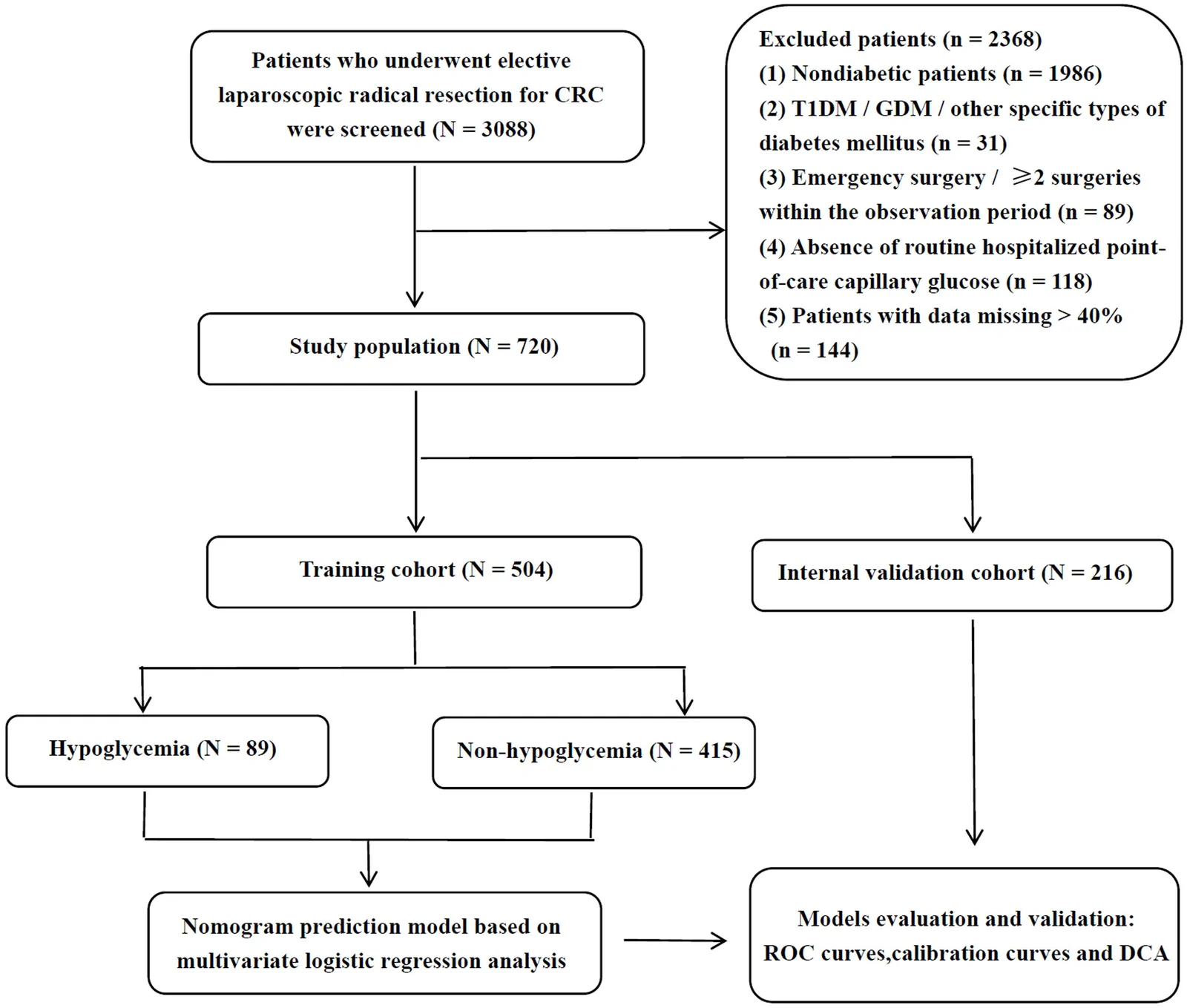
Flowchart diagram of the study CRC, colorectal cancer; T1DM, type 1 diabetes mellitus; GDM, gestational diabetes mellitus; ROC, receiver operating characteristic; DCA, decision curve analysis.

A total of 3088 patients undergoing elective laparoscopic CRC resection during the study period, and 720 patients were included based on the inclusion and exclusion criteria (**Figure 1**). Of the overall cohort, 387 participants (53.8%) were male, with the median age of 67.00 years and the median T2DM disease duration of 1.50 years. Among them, 120 patients experienced at least one hypoglycemic event during their inpatient stay after laparoscopic CRC resection, yielding an incidence of 16.7%. Of these patients, 73.3% developed hypoglycemia within the first postoperative week. The incidence of level 1 and level 2 hypoglycemia were 13.6% (98/720) and 3.1% (22/720), respectively.

These patients were then randomly assigned to the training (504 cases) and internal validation (216 cases) cohorts. **Table 1** summarized the baseline characteristics of the included patients.

**Table 1:** Comparison of baseline features between the training and internal validation cohort.

Characteristic	Total N = 720	Training cohort			Internal validation cohort		
		Total N = 504	Hypoglycemia N = 89	Non-hypoglycemia N = 415	Total N = 216	Hypoglycemia N = 31	Non-hypoglycemia N = 185
Demographic characteristics							
Age, y	67.00 (60.00, 73.00)	67.00 (60.00, 73.00)	68.00 (64.00, 75.00)	67.00 (59.00, 73.00)	67.00 (60.00, 74.00)	74.00 (68,.00 80.00)	66.00 (59.00, 73.00)
Sex, n, male	387 (53.8%)	277 (55.0%)	38 (42.7%)	239 (57.6%)	110 (50.9%)	13 (41.9%)	97 (52.4%)
BW, kg	59.00 (52.00, 66.00)	59.50 (52.00, 67.00)	58.00 (50.00, 65.00)	60.00 (52.00, 68.00)	59.00 (51.00, 65.00)	56 (46, 62)	60 (52, 67)
BMI, kg/m^2^	22.51 (20.44, 24.77)	22.49 (20.31, 24.64)	21.78 (19.85, 23.47)	22.65 (20.42, 24.77)	22.67 (20.62, 25.00)	21.45 (18.42, 23.01)	22.89 (20.85, 25.51)
T2DM duration, y	1.50 (1.00, 11.00)	1.50 (1.00, 11.00)	2.50 (1.00, 13.00)	1.00 (1.00, 11.00)	1.50 (1.00, 12.00)	21.50 (18.40, 23.00)	22.90 (20.90, 25.50)
Smoke, yes	107 (14.86%)	77 (15.3%)	11 (12.4%)	66 (15.9%)	30 (13.9%)	3 (9.7%)	27 (14.6%)
Drink, yes	28 (3.89%)	21 (4.2%)	3 (3.4%)	18 (4.3%)	7 (3.2%)	2 (6.5%)	5 (2.7%)
Comorbidities							
Hypertension, yes	186 (25.83%)	127 (25.2%)	23 (25.8%)	104 (25.1%)	59 (27.3%)	12 (38.7%)	47 (25.4%)
Hyperlipidemia, yes	17 (2.36%)	12 (2.4%)	1 (1.1%)	11 (2.7%)	5 (2.3%)	0 (0.0%)	5 (2.7%)
Thyroid disease, yes	26 (3.61%)	20 (4.0%)	5 (5.6%)	15 (3.6%)	6 (2.8%)	1 (3.2%)	5 (2.7%)
Renal failure, yes	29 (4.03%)	21 (4.2%)	8 (9.0%)	13 (3.1%)	8 (3.7%)	3 (9.7%)	5 (2.7%)
Baseline laboratory tests							
HbA1c^a^, %	7.05 (6.19, 8.24)	7.05 (6.17, 8.22)	7.05 (6.07, 8.32)	7.05 (6.18, 8.22)	6.98 (6.23, 8.29)	6.85 (6.07, 8.15)	6.99 (6.23, 8.29)
eGFR^a^, mL/(min·1.73m^2^)	62.18 (47.50, 77.99)	61.56 (47.51, 77.61)	52.99 (41.22, 71.66)	62.42 (49.30, 78.44)	63.87 (47.48, 80.93)	49.05 (31.36, 70.51)	65.51 (49.64, 82.99)
ALT^a^, U/L	23.68 (14.58, 36.05)	24.19 (14.41, 36.59)	25.00 (14.13, 33.66)	23.22 (14.39, 37.78)	22.74 (16.69, 33.29)	23.02 (15.17, 34.59)	22.88 (15.17, 34.59)
AST^a^, U/L	20.23 (15.34, 29.40)	20.14 (15.40, 29.18)	21.00 (16.78, 29.30)	19.80 (15.34, 29.23)	20.44 (15.15, 29.88)	22.19 (18.85, 31.79)	19.65 (15.15, 29.78)
TC^a^, mmol/L	5.00 (3.86, 5.94)	5.01 (3.85, 6.00)	4.91 (4.07, 5.91)	5.01 (3.84, 6.02)	4.93 (3.96, 5.84)	4.61 (4.23, 5.47)	5.01 (3.86, 5.86)
TG^a^, mmol/L	1.92 (1.29, 2.84)	1.91 (1.23, 2.75)	1.96 (1.14, 2.97)	1.89 (1.23, 2.73)	1.95 (1.35, 3.19)	1.66 (1.32, 2.71)	1.99 (1.38, 3.19)
HDL-C^a^, mmol/L	1.04 (0.92, 1.26)	1.04 (0.92, 1.24)	0.97 (0.87, 1.17)	1.05 (0.93, 1.26)	1.05 (0.93, 1.30)	1.05 (0.91, 1.29)	1.04 (0.93, 1.30)
LDL-C^a^, mmol/L	2.98 (2.30, 3.62)	3.00 (2.29, 3.63)	3.01 (2.30, 3.70)	3.00 (2.27, 3.58)	2.89 (2.34, 3.57)	2.91 (2.42, 3.49)	2.89 (2.33, 3.57)
FPG, mmol/L (the day prior to operation)	6.50 (5.41, 8.01)	6.45 (5.40, 8.01)	5.90 (5.07, 7.00)	6.60 (5.48, 8.21)	6.60 (5.49, 8.00)	6.60 (5.30, 8.84)	6.70 (5.50, 7.88)
2hPPG, mmol/L (the day prior to operation)	7.88 (6.20, 10.19)	7.90 (6.31, 10.20)	7.70 (6.40, 9.32)	8.00 (6.30, 10.45)	7.70 (5.86, 10.02)	8.80 (5.40, 10.63)	7.50 (5.88, 10.02)
MPG, mmol/L	7.76 (6.60, 9.19)	7.76 (6.66, 9.18)	7.42 (6.33, 9.31)	7.92 (6.68, 9.16)	7.73 (6.45, 9.20)	7.52 (6.44, 9.02)	8.50 (6.71, 11.41)
Perioperative data							
Nutritional support							
EN	97 (13.47%)	71 (14.1%)	16 (18.0%)	55 (13.3%)	26 (12.0%)	5 (16.1%)	21 (11.4%)
EN+PN	104 (14.44%)	79 (15.7%)	19 (21.3%)	60 (14.5%)	25 (11.6%)	2 (6.5%)	23 (12.4%)
No	157 (21.81%)	247 (49.0%)	36 (40.4%)	211 (50.8%)	115 (53.2%)	18 (58.1%)	97 (52.4%)
PN	362 (50.28%)	107 (21.2%)	18 (20.2%)	89 (21.4%)	50 (23.1%)	6 (19.4%)	44 (23.8%)
Operation duration, min	155.00 (140.00, 175.00)	155.00 (140.00, 180.00)	160.00 (147.00, 180.00)	155.00 (140.00, 180.00)	155.00 (140.00, 175.00)	160.00 (138.00, 175.00)	155.00 (140.00, 170.00)
Anesthetic mode							
general anesthesia	629 (87.36%)	440 (87.3%)	78 (87.6%)	362 (87.2%)	189 (87.5%)	30 (96.8%)	159 (85.9%)
general anesthesia and CSEA	48 (6.67%)	33 (6.5%)	5 (5.6%)	28 (6.7%)	15 (6.9%)	0 (0.0%)	15 (8.1%)
general anesthesia and nerve block anesthesia	43 (5.97%)	33 (6.5%)	6 (6.7%)	25 (6.0%)	12 (5.6%)	1 (3.2%)	11 (5.9%)
ASA grade > II	344 (47.78%)	234 (46.4%)	56 (62.9%)	178 (42.9%)	110 (50.9%)	21 (67.7%)	89 (48.1%)
Postoperative fasting time, d	4.00 (1.00, 7.00)	4.00 (2.00, 7.00)	5.00 (2.00, 9.00)	4.00 (2.00, 7.00)	4.00 (1.00, 7.00)	6.00 (2.00, 8.00)	3.00 (1.00, 7.00)
PLOS, d	5.00 (3.00, 8.00)	6.00 (3.00, 8.00)	6.00 (3.00, 8.00)	5.00 (3.00, 8.00)	5.00 (3.00, 8.00)	7.00 (2.00, 11.00)	5.00 (3.00, 8.00)
Inflammatory parameters on POD 3							
CRP, mg/L	100.00 (70.33, 127.00)	101.00 (69.76, 128.34)	108.20 (68.82, 150.50)	98.43 (69.76, 126.00)	97.89 (74.28, 126.00)	111.00 (80.80, 136.00)	94.50 (73.80, 125.07)
WBC, 10^9^/L	8.54 (6.43, 11.48)	8.48 (6.42, 12.00)	8.43 (5.82, 13.31)	8.48 (6.59, 11.91)	8.61 (6.54, 10.76)	7.98(6.56, 9.97)	8.92 (6.46, 11.09)
NEUT, %	81.02 (74.36, 85.87)	81.04 (74.18, 85.97)	83.17 (74.98, 86.89)	80.98 (74.09, 85.67)	80.82 (74.90, 85.76)	85.41 (77.79, 89.78)	80.62 (73.39, 85.34)
Other perioperative medications							
Diuretic, yes	24 (3.33%)	19 (3.8%)	6 (6.7%)	13 (3.1%)	5 (2.3%)	1 (3.2%)	4 (2.2%)
β-blocker, yes	27 (3.75%)	19 (3.8%)	5 (5.6%)	14 (3.4%)	8 (3.7%)	1 (3.2%)	7 (3.8%)
Somatostatin, yes	62 (8.61%)	46 (9.1%)	10 (11.2%)	36 (8.7%)	16 (7.4%)	3 (9.7%)	13 (7.0%)
Quinolones, yes	14 (1.94%)	11 (2.2%)	3 (3.4%)	8 (1.9%)	3 (1.4%)	0 (0.0%)	3 (1.6%)
Previous prolonged insulin treatment^b^, yes	181 (25.14%)	130 (25.8%)	37 (41.6%)	93 (22.4%)	51 (23.6%)	10 (32.3%)	41 (22.2%)
Postoperative insulin administration^c^, yes	300 (41.67%)	215 (42.7%)	57 (64.0%)	158 (38.1%)	85 (39.4%)	16 (51.6%)	69 (37.3%)

Cases (n, percentage/%) or median (interquartile range).

^a^ Testing on admission.

^b^ Preoperative continuation of pre-admission regimens. Preoperative insulin regimens in the training cohort: hypoglycemia group: premixed insulin (N = 25), rapid-acting plus long-acting insulin (N = 12), and long-acting insulin alone (N = 1); non-hypoglycemia group: premixed insulin (N = 76), rapid-acting plus long-acting insulin (N = 15), and long-acting insulin alone (N = 2).

^c^ Postoperative insulin regimens in the training cohort: hypoglycemia group: premixed insulin (N = 11), rapid-acting plus long-acting insulin (N = 39), and long-acting insulin alone (N = 7); non-hypoglycemia group: premixed insulin (N = 26), rapid-acting plus long-acting insulin (N = 121), and long-acting insulin alone (N = 11).

BW, body weight; BMI, body mass index; T2DM, type 2 diabetes mellitus; HbA1c, glycated hemoglobin; eGFR, estimated glomerular filtration rate; ALT, alanine transaminase; AST, aspartate transaminase; TC, total cholesterol; TG, triglyceride; HDL-C, high density lipoprotein cholesterol; LDL-C, low density lipoprotein cholesterol; FPG, fasting plasma glucose; 2hPPG, 2-hour postprandial plasma glucose; MPG, 3-day preoperative mean plasma glucose; EN, enteral nutrition; PN, parenteral nutrition; CSEA, combined spinal epidural anesthesia; ASA, American Society of Anesthesiologists; PLOS, postoperative length of stay; POD, postoperative day; CRP, c reactive protein; WBC, white blood cell; NEUT, neutrophil

### Univariate analysis of risk factors for postoperative hypoglycemia

Univariate comparisons of risk factors between the hypoglycemia and non-hypoglycemia patients in the training cohorts were outlined in **Supplementary Table 2**. There were significant intergroup differences in age, gender, T2DM duration, complicated with renal failure, baseline eGFR, FPG (the day prior to operation), ASA grade > II, postoperative fasting time, previous prolonged insulin treatment, and postoperative insulin administration (all *p* values < 0.05).

### Multivariate logistic regression analysis of risk factors for postoperative hypoglycemia

Complicated with renal failure was associated with eGFR values (*r* = 0.210, *p* = 0.005). A robust correlation was identified between previous prolonged insulin treatment and postoperative insulin administration (*r* = 0.514, *p* < 0.001). We further quantified collinearity by computing the VIF for each variable. eGFR (VIF = 21.432) and postoperative insulin administration (VIF = 17.921) both returned VIF scores well above 10 (**Supplementary Table 3**), confirming the presence of severe multicollinearity. Both eGFR and postoperative insulin administration were not entered into the logistic regression model.

Logistic regression analysis demonstrated that long T2DM duration (odds ratio [OR], 1.05; 95% confidence interval [CI], 1.01-1.09), low preoperative day-1 FPG (OR, 0.72; 95%CI, 0.62-0.84), ASA grade > II (OR, 10.51; 95%CI, 4.06-28.02), long postoperative fasting (OR, 1.10; 95%CI, 1.04-1.16), and previous prolonged insulin treatment (OR, 16.42; 95%CI, 5.88-45.82) were independent risk factors for postoperative hypoglycemia in T2DM patients undergoing laparoscopic CRC resection (**Table 2**).

**Table 2:** Multivariate logistic regression analysis of predictors for hypoglycemia following laparoscopic CRC resection in hospitalized patients with T2DM in the training cohort.

Variables	β	SE	Wald	OR	95% CI	*p*
T2DM duration	0.06	0.02	11.39	1.05	1.01, 1.09	0.007
FPG (the day prior to operation)	-0.22	0.07	12.65	0.72	0.62, 0.84	< 0.001
ASA grade > II	2.37	0.49	23.28	10.51	4.06, 28.02	< 0.001
Postoperative fasting time	0.07	0.02	8.89	1.10	1.04, 1.16	< 0.001
Previous prolonged insulin treatment	2.80	0.52	28.57	16.42	5.88-45.82	< 0.001
Constant	-2.86	1.60	3.18	0.057	/	0.075

CRC, colorectal cancer; T2DM, type 2 diabetes mellitus; β: regression coefficient; SE: standard error; OR, odds ratio; CI, confidence interval; FPG, fasting plasma glucose; ASA, American Society of Anesthesiologists.

**Figure 2:**
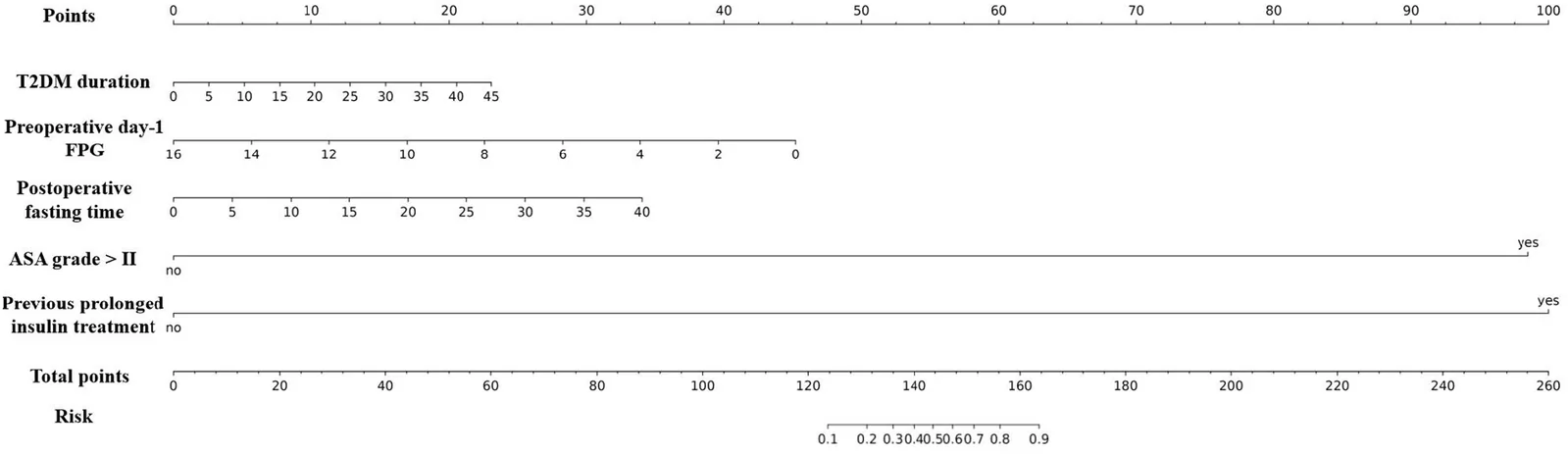
Nomogram for predicting postoperative hypoglycemia in hospitalized patients with T2DM undergoing laparoscopic CRC resection in the training cohort CRC, colorectal cancer; T2DM, type 2 diabetes mellitus; FPG, fasting plasma glucose; ASA, American Society of Anesthesiologists.

Based on multivariable logistic regression results, a nomogram was established to estimate postoperative hypoglycemia risk in T2DM patients during the perioperative period of laparoscopic CRC resection, as illustrated in **Figure 2**.

### The performance of training and internal validation cohorts

**Figure 3:**
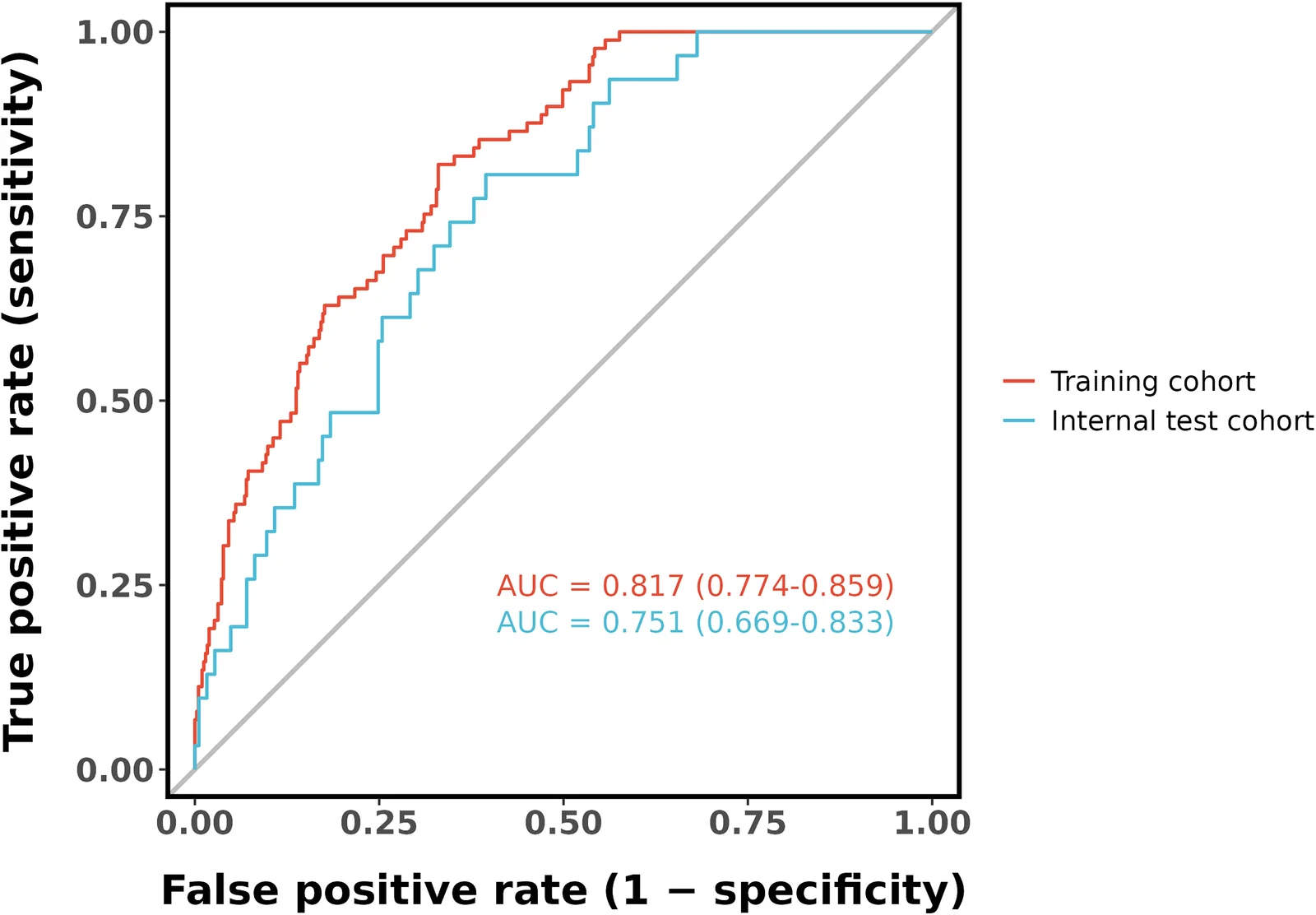
The receiver operating characteristic curve and area under the receiver operating characteristic curve for the prediction of hypoglycemia after laparoscopic CRC resection in hospitalized patients with T2DM CRC, colorectal cancer; T2DM, type 2 diabetes mellitus; AUC, area under the curve.

The model yielded an AUC of 0.817 (95% CI, 0.774-0.859) in the training cohort and 0.751 (95% CI, 0.669-0.833) in the internal validation cohort for postoperative hypoglycemia prediction (**Figure 3**), indicating satisfactory discriminatory power in the validation set. With a bootstrap-corrected concordance index of 0.803 (95% CI 0.761-0.845), the nomogram exhibited acceptable internal validity, yet external validation was still warranted to confirm its performance in independent cohorts.

**Figure 4:**
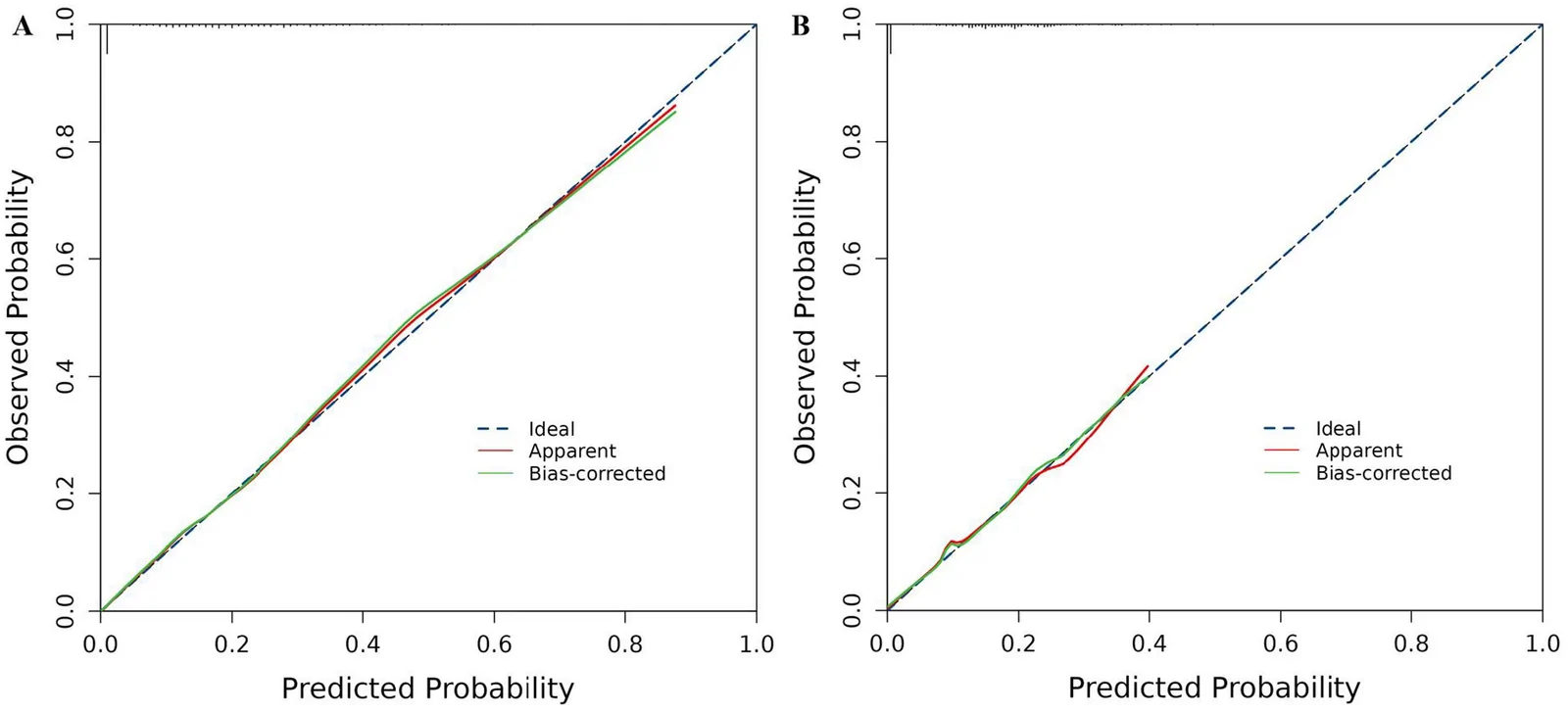
Calibration curve for the prediction of hypoglycemia after laparoscopic CRC resection in hospitalized patients with T2DM. **(A)** Development cohort; **(B)** Validation cohort CRC, colorectal cancer; T2DM, type 2 diabetes mellitus.

**Figure 5:**
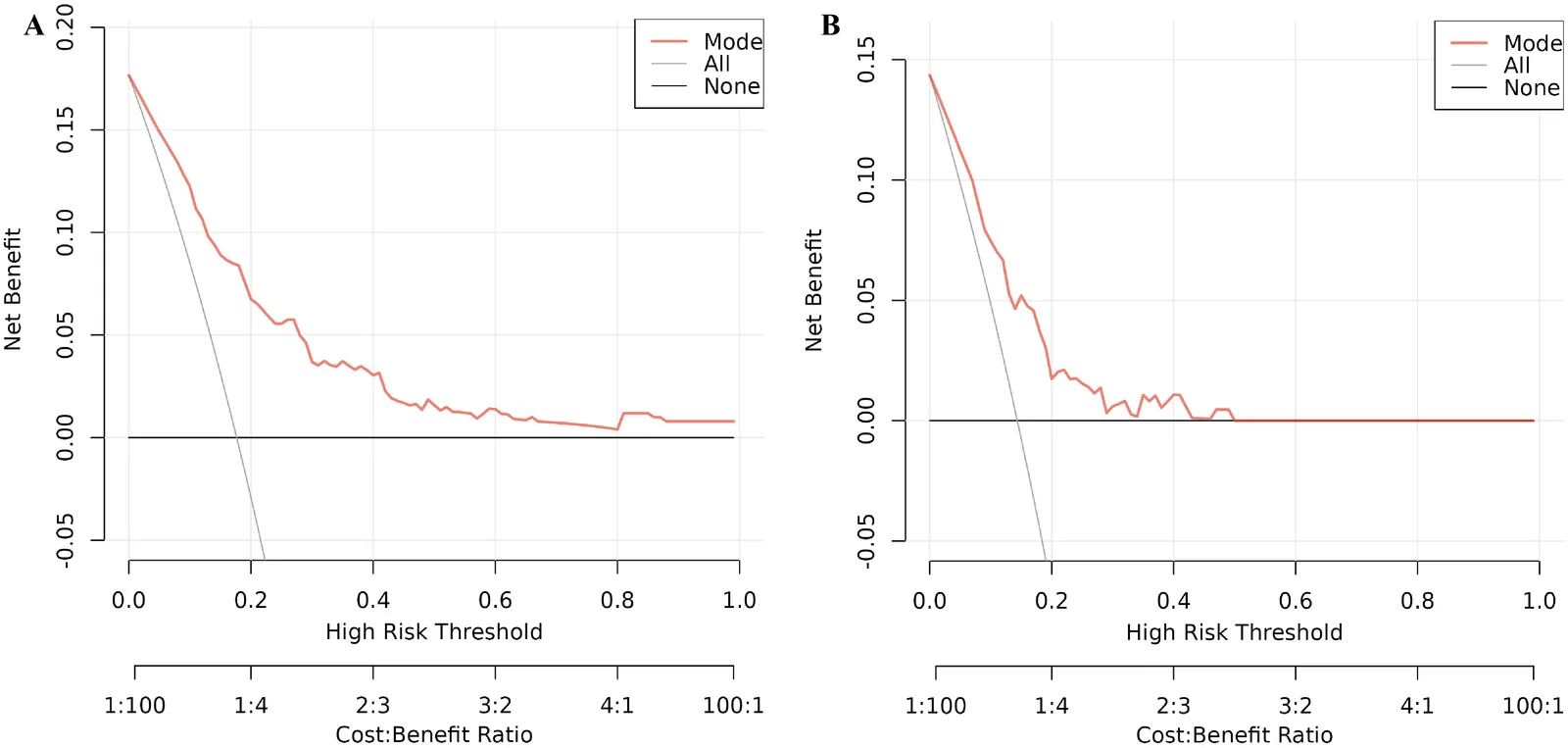
Decision curve analysis for predicting hypoglycemia after laparoscopic CRC resection in hospitalized patients with T2DM. **(A)** Development cohort; **(B)** Validation cohort. CRC, colorectal cancer; T2DM, type 2 diabetes mellitus.

Calibration curves showed good concordance between predicted and observed probabilities in both cohorts (**Figure 4**), with Hosmer-Lemeshow test *p* values of 0.914 (χ² = 3.307) and 0.802 (χ² = 2.962) for training and internal validation cohorts, respectively. In the training cohort, the model yielded a calibration slope of 1.000, a calibration intercept of 0.000, and a Brier score of 0.115. The corresponding performance metrics in the internal validation cohort were 1.000, 0.000, and 0.105, respectively. Decision curve analysis demonstrated that the model’s curve lay above both the none line and the all line at threshold probabilities between 6% and 85% (**Figure 5**), indicating favorable clinical utility and substantial net benefit for patients. In the internal validation cohort, for example, a threshold probability of 18% on the horizontal axis yielded a corresponding vertical axis value of roughly 0.05, translating to around 5 per 100 individuals benefiting from the model at this risk cutoff.

### Analysis of risk factors for postoperative level 1 and level 2 hypoglycemia

Results of univariable risk factor analysis for level 1 and level 2 hypoglycemia groups were presented in (**Supplementary Tables 3**, **4**). In subsequent analyses, multivariable regression was rerun separately in the entire cohort for the two hypoglycemia subtypes: level 1 (n = 98) and level 2 (n = 22). Factors independently associated with postoperative level 1 hypoglycemia were long T2DM duration (OR, 1.05; 95%CI, 1.02-1.09), low preoperative day-1 FPG (OR, 0.78; 95%CI, 0.69-0.89), and long postoperative fasting (OR, 1.07; 95%CI, 1.01-1.12). On the other hand, On the other hand, low BMI (OR, 0.55; 95%CI, 0.33-0.90), low preoperative day-1 FPG (OR, 0.31; 95%CI, 0.11-0.92), and high postoperative day-3 NEUT proportion (OR, 1.22; 95%CI, 1.03-1.44) were found to be associated with postoperative level 2 hypoglycemia. Both these two models exhibited favorable calibration performance (**Table 3**). Nomograms were constructed to graphically represent the combined contribution of multiple incorporated risk factors, as detailed in **Supplementary Figures 1**, **2**.

**Table 3:** Multivariate logistic regression analysis of predictors for level 1 and level 2 hypoglycemia following laparoscopic CRC resection in hospitalized patients with T2DM^a^.

Hypoglycemia classification	Variables	β	SE	Wald	OR	95% CI	*p*
Level 1 hypoglycemia^bd^ N = 98	T2DM duration	0.05	0.02	8.59	1.05	1.02, 1.09	0.001
	FPG (the day prior to operation)	-0.24	0.06	14.39	0.78	0.69, 0.89	< 0.001
	Postoperative fasting time	0.03	0.01	5.11	1.07	1.01, 1.12	0.017
	Constant	-2.37	1.32	3.23	0.093		0.072
Level 2 hypoglycemia^ce^ N = 22	BMI	-0.50	0.16	9.32	0.55	0.33, 0.90	0.018
	FPG (the day prior to operation)	-0.65	0.27	5.58	0.31	0.11, 0.92	0.034
	NEUT	0.15	0.06	6.21	1.22	1.03, 1.44	0.019
	Constant	-1.56	5.40	0.08	0.21		0.772

CRC, colorectal cancer; T2DM, type 2 diabetes mellitus; OR, odds ratio; CI, confidence interval; FPG, fasting plasma glucose; BMI, body mass index; FPG, fasting plasma glucose.

^a^ Patients with both level 1 and level 2 postoperative hypoglycemia were classified exclusively as level 2 hypoglycemia cases.

^b^ The final analytical cohort for level 1 hypoglycemia comprised 698 cases [total cases (720) minus 22 patients with level 2 hypoglycemia].

^c^ The final analytical cohort for level 2 hypoglycemia comprised 622 cases [total cases (720) minus 98 patients with level 1 hypoglycemia].

^d^ AUC in the ROC analysis: 0.821 (95% CI, 0.776-0.867); Hosmer-Lemeshow test *p* values: 0.801 (χ² = 4.586); calibration slope: 1.000; calibration intercept: 0.000; Brier score: 0.084.

^e^ AUC in the ROC analysis: 0.886 (95% CI, 0.839-0.934); Hosmer-Lemeshow test *p* values: 0.966 (χ² = 2.411); calibration slope: 1.000; calibration intercept: 0.000; Brier score: 0.010.

## Discussion

In this cohort study, a nomogram was constructed to predict the risks of postoperative hypoglycemia in hospitalized patients with T2DM undergoing laparoscopic CRC resection. To our knowledge, this is the first predictive model for hypoglycemia among T2DM patients following a single surgical procedure. In contrast to the common published perioperative hypoglycemia prediction models, this single-procedure inpatient model might help enabling targeted prevention and control of postoperative hypoglycemia. Validation of the nomogram demonstrated good discriminative performance and calibration accuracy. DCA confirmed the clinical utility of the nomogram over a wide spectrum of risk thresholds. The final prediction model incorporated five independent predictors: T2DM duration, preoperative day-1 FPG level, ASA physical status classification, postoperative fasting period, and preoperative long-term insulin therapy. Additionally, this study preliminarily identified specific risk factors for both level 1 and level 2 postoperative hypoglycemia.

Hypoglycemia following gastrointestinal surgery arises from a multifactorial pathophysiological process (24). A primary driver is the anatomical restructuring of the digestive tract, which leads to rapid gastric emptying. Moreover, gastrointestinal surgery is frequently associated with augmented GLP-1 release, an effect that may provoke disproportionate insulin secretion and precipitate hypoglycemic events. Compounding these effects are a range of additional factors: enhanced insulin sensitivity following surgical intervention, aberrant parasympathetic nervous system regulation, compositional changes in the intestinal microbiome, and dysregulated serotonin metabolism.

The overall postoperative hypoglycemia incidence in our cohort was 16.7%, higher than the 12.6% rate documented in a previous study of patients undergoing gastrointestinal surgery (19). In addition to the potential differences in sample size and surgical settings, this discrepancy may be attributed to the marked impact of CRC surgery on intestinal function (25). Postoperative dietary recovery after CRC surgery requires bowel function return and flatus passage, which may be prolonged compared with other gastrointestinal surgeries.

The present study identified a positive association between T2DM duration and postoperative hypoglycemia after laparoscopic CRC resection, consistent with prior findings in other surgical populations (18, 19). Pancreatic β-cell function exhibits a time-dependent progressive decline after diabetes onset, largely driven by the loss of β-cell mass (26). The United Kingdom Prospective Diabetes Study (UKPDS) reported a 5% annual decline in β-cell function post-T2DM diagnosis, while a 2% annual rate was demonstrated in Chinese T2DM patients (27). A marked decline in pancreatic β-cell function impairs the regulation of insulin and counterregulatory hormones, increasing glycemic variability (GV).

Intensive perioperative glycemic control may be associated with a 3.36-fold higher risk of postoperative hypoglycemia compared to conventional glycemic control (10). In this cohort analysis, preoperative day-1 FPG was significantly lower in the hypoglycemia group and identified as an independent risk factor for postoperative hypoglycemia, consistent with findings by ZX Liu et al. (11) across all elective surgeries. The optimal preoperative glycemic level for T2DM patients undergoing laparoscopic CRC resection remains understudied. Despite ongoing controversy, intensive period- ergative glucose control may reduce cardiovascular event risk but not all-cause mortality or postoperative infections in surgical T2DM patients versus conventional glycemic control (10). Therefore, a moderate guideline-based preoperative glycemic target (28) may be preferable for T2DM patients without cardiovascular comorbidities undergoing laparoscopic CRC resection.

As a foundational risk stratification instrument in anesthesiology, the ASA physical status classification system is principally designed to characterize patients’ overall physiological condition and quantify the perioperative risks associated with surgical procedures and anesthesia administration. Among this enrolled T2DM cohort, the distribution of ASA physical status classification spanned grades II through IV, with ASA grade > II being an independent risk factor for post-CRC hypoglycemia. Riegger LQ et al. (29) reported a significant association between ASA grade ≥ III and higher intraoperative hypoglycemia risk among pediatric surgical cohorts. Elevated ASA physical status classification is postulated to correlate with impaired organ compensatory function, increased glucose consumption, and diminished gluconeogenic capacity. However, given that available evidence was predominantly derived from pediatric populations, the ASA grade-postoperative hypoglycemia association has yet to be corroborated by targeted investigations in adult surgical populations.

In this patient cohort, no routine preoperative fasting protocol was implemented prior to laparoscopic CRC resection. The duration of postoperative fasting ranged from 1 to 20 days. Accounting for the physiological impacts of surgical trauma, intra-abdominal inflammation and an aesthetic exposure, maintaining fasting status following CRC resection is traditionally viewed as effective for alleviating the risk of postoperative gastrointestinal adverse events including nausea, vomiting and abdominal distension. However, extended periods of fasting places the body in a catabolic metabolic state, thereby potentially elevating GV (30). Prolonged postoperative fasting was found to be independently associated with postoperative hypoglycemia in T2DM patients undergoing laparoscopic CRC resection in the present study. The optimal timing of postoperative feeding initiation remains incompletely defined. Nevertheless, extensive evidence demonstrated that early reintroduction of oral nutrition after laparoscopic CRC resection expedited the restoration of gastrointestinal function, improved nutrient delivery, and shortened hospital stay, with no concomitant elevation in postoperative morbidity risk (31, 32).

Insulin therapy constitutes a major pharmacologic risk factor for hypoglycemia among T2DM populations. It has been consistently documented as a predictor of perioperative hypoglycemic events across other surgical cohorts in existing literature (18, 19), an observation further corroborated by data from the present study. Within the inpatient T2DM population, subcutaneous insulin administration might be associated with a 4-fold increase in the risk of hypoglycemia (33). In this patient cohort, preoperative antihyperglycemic therapy was maintained in line with each patient’s established long-term outpatient prescription regimen. Subcutaneous insulin injection is one of the primary measures for hyperglycemia in patients with impaired pancreatic β-cell function recommended by current clinical guidelines (28). In the present study, pre-existing long- term insulin regimens were maintained for preoperative glycemic management. This practice served as an indirect marker of diminished pancreatic reserve among study subjects; and as noted aforementioned, this further increased the risk of GV. Perioperative delivery of continuous subcutaneous insulin infusion (CSII), combined with continuous glucose monitoring (CGM), may effectively reduce hypoglycemia in this patient population.

Our stratified analysis tentatively identified the independent predictors of level 1 and 2 postoperative hypoglycemia after laparoscopic CRC resection, facilitating the implementation of targeted preventive measures tailored to hypoglycemia severity. The association between low BMI and severe hypoglycemia in T2DM has been widely validated in prior research (34). Lower BMI partially reflects malnutrition, a known contributor to increased hypoglycemia risk. Notably, high circulating insulin- like growth factor-1 (IGF-1) expression in CRC, coupled with low BMI-driven IGF-1 elevation, may trigger hypoglycemia (35). On the other hand, abrupt drops in circulating glucose levels can prompt a rapid catecholamine surge and elicit a systemic inflammatory cascade (36). Hypoglycemia-related pro-inflammatory mechanisms were reportedly linked to worse prognosis in acute myocardial infarction (37). Therefore, individualized perioperative glycemic control targets should be established for patients with low BMI. Additionally, close perioperative blood counts monitoring may aid in the timely detection of potential hypoglycemic risks and support corresponding adjustments to glucose-lowering treatment regimens.

What’s more, age has been well documented as a predictor of some adverse outcomes after CRC resection, including postoperative surgical site infections and pulmonary infections (38, 39). Unexpectedly, age failed to serve as an independent predictor of postoperative hypoglycemia in our study, despite a significantly higher mean age in the hypoglycemia group relative to the non-hypoglycemia group in the training cohort. This discrepancy might be attributed to the limited sample size of our study, as well as the effects of perioperative stress and surgery-induced blood glucose fluctuations. Concordantly, several previous studies focused on predictive models for perioperative hypoglycemia did not detect age as an independent risk factor in the perioperative population (11, 18, 19).

The present study had some limitations. First, while preoperative models guide perioperative assessment, CRC surgery raises hypoglycemia risk via prolonged fasting and nutritional support. This study only preliminarily established an in-hospital hypoglycemia prediction model after laparoscopic CRC resection. Notably, only preliminary model development was conducted here, with no external validation to date, leaving its generalizability to other centers and patient cohorts unconfirmed. Significantly elevated adjusted ORs were observed for ASA grade > II (OR 10.51) and prior insulin treatment (OR 16.42). This might stem from the relatively small sample size inflating variable-outcome associations. Additional studies with larger cohorts or multicenter data are needed for further confirmation. Third, as the continuous variables under investigation were important recognized confounders requiring statistical adjustment, the absence of formal linearity evaluation in this study might result in prediction bias and undermining the overall predictive performance of the regression model. Screening variables by univariate statistical significance before constructing a multivariable logistic regression model risked omitting clinically relevant predictive factors. The relatively low incidence of level 2 hypoglycemia might potentially impair corresponding model stability. Fourth, this retrospective EMRs analysis omitted potential factors (C-peptide, infusion volume, nutrition, mood, sleep, and physical activity), which might bias the findings. This model could not account for finger-stick glucose test errors. Finally, scarce researches on laparoscopic CRC resection perioperative glycemic regulation prevented full analysis of these risk factors’ links to surgery.

In summary, the nomogram developed in this study demonstrated good predictive performance for hypoglycemia in hospitalized T2DM patients after laparoscopic CRC resection; and further identified predictive factors for varying degrees of hypoglycemia. It achieved acceptable predictive accuracy without reliance on CGM data, constituting a clinically valuable reference for routine practice. Prospective validation was warranted to examine the real-time performance of model-driven informatics alerts in mitigating the incidence of this clinically significant adverse event.

## Data Availability

The original raw data are available from the corresponding author upon reasonable request.
