# Supplementary material for: Development and validation of a hypoglycemia risk prediction model for hospitalized type 2 diabetes patients following laparoscopic colorectal cancer resection: a retrospective cohort study
Source: Front Endocrinol (Lausanne). 2026 Aug 28;17:1932701. doi: 10.3389/fendo.2026.1932701 (PMC13561915; doi:10.3389/fendo.2026.1932701)
Supplement: Supplementary file 1 [file SupplementaryFile1.docx]

Supplementary Material

**TABLE S1** Characteristics of variables with missing data in the overall dataset and sensitivity analysis via comparison between post-imputation and original datasets

|  | Number (%) with  missing data | Complete case  (*χ^2^/P*) | Multiple imputation  (*χ^2^/P*) |
| --- | --- | --- | --- |
| BMI | 5(0.69%) | 3.483/0.059 | 3.452/0.060 |
| HbA1c | 4 (0.56%) | 3.063/0.085 | 3.564/0.066 |
| CRP | 8(1.11%) | 1.120/0.288 | 1.843/0.192 |

BMI, body mass index; HbA1c, glycated haemoglobin; CRP, c reactive protein

**TABLE S2** Univariate analysis of predictors for hypoglycemia following laparoscopic CRC resection in hospitalized patients with T2DM in the training cohort

| Variables | Univariate analysis | |
| --- | --- | --- |
|  | Crude OR (95%CI) | *p* |
| Age | 1.03 (1.00, 1.05) | 0.020 |
| Male | 0.55 (0.35, 0.87) | 0.011 |
| BW | 0.99 (0.97, 1.01) | 0.297 |
| BMI | 0.94 (0.88, 1.01) | 0.080 |
| T2DM duration | 1.03 (1.00, 1.06) | 0.046 |
| Smoke | 0.75 (0.38, 1.48) | 0.400 |
| Drink | 0.77 (0.22, 2.67) | 0.680 |
| Complicated with hypertension | 1.04 (0.62, 1.76) | 0.877 |
| Complicated with hyperlipidemia | 0.42 (0.05, 3.27) | 0.406 |
| Complicated with thyroid disease | 1.59 (0.56, 4.49) | 0.383 |
| Complicated with renal failure | 3.05 (1.23, 7.61) | 0.016 |
| HbA1c | 1.10 (0.95, 1.26) | 0.196 |
| eGFR | 0.98 (0.97, 0.99) | < 0.001 |
| ALT | 1.00 (0.99, 1.01) | 0.937 |
| AST | 1.01 (0.99, 1.02) | 0.331 |
| TC | 0.96 (0.83, 1.12) | 0.646 |
| TG | 1.02 (0.92, 1.13) | 0.706 |
| HDL-C | 0.67 (0.32, 1.42) | 0.293 |
| LDL-C | 1.17 (0.92, 1.48) | 0.207 |
| FPG  (the day prior to operation) | 0.79 (0.70, 0.90) | < 0.001 |
| 2HPPG  (the day prior to operation) | 0.98 (0.91, 1.06) | 0.637 |
| MPG | 0.99 (0.88, 1.11) | 0.840 |
| Operation duration | 1.01 (1.00, 1.02) | 0.099 |
| Anesthetic mode |  |  |
| general anesthesia | 1.04 (0.52, 2.08) | 0.916 |
| general anesthesia and CSEA | 0.83 (0.31, 2.21) | 0.708 |
| general anesthesia and  nerve block anesthesia | 1.11 (0.44, 2.81) | 0.819 |
| ASA grade > Ⅱ | 2.26 (1.41, 3.62) | < 0.001 |
| Postoperative fasting time | 1.06 (1.02, 1.10) | 0.003 |
| PLOS | 1.02 (0.98, 1.06) | 0.372 |
| CRP | 1.00 (1.00, 1.00) | 0.603 |
| WBC | 1.01 (0.95, 1.06) | 0.829 |
| NEUT | 1.02 (0.99, 1.04) | 0.196 |
| Diuretic | 2.24 (0.83, 6.05) | 0.113 |
| β-blocker | 1.70 (0.60, 4.86) | 0.318 |
| Somatostatin | 1.33 (0.63, 2.80) | 0.448 |
| Quinolones | 1.77 (0.46, 6.83) | 0.404 |
| Previous prolonged insulin treatment | 2.46 (1.52, 3.98) | < 0.001 |
| Postoperative insulin administration | 2.90 (1.80, 4.66) | < 0.001 |

CRC, colorectal cancer; T2DM, type 2 diabetes mellitus; OR, odds ratio; CI, confidence interval; BW, body weight; BMI, body mass index; HbA1c, glycated haemoglobin; eGFR, estimated glomerular filtration rate; ALT, alanine transaminase; AST, aspartate transaminase; TC, total cholesterol; TG, triglyceride; HDL-C, high density lipoprotein cholesterol; LDL-C, low density lipoprotein cholesterol; FPG, fasting plasma glucose; 2hPPG, 2-hour postprandial plasma glucose; MPG, 3-day preoperative mean plasma glucose; EN, enteral nutrition; PN, parenteral nutrition; CSEA, combined spinal epidural anesthesia; ASA, American Society of Anesthesiologists; PLOS, postoperative length of stay; POD, postoperative day; CRP, c reactive protein; WBC, white blood cell; NEUT, neutrophil

**TABLE S3** Variance inflation factor and tolerance

| Variables | VIF | tolerance |
| --- | --- | --- |
| Age | 2.226 | 0.449 |
| Sex | 4.110 | 0.159 |
| BW | 3.891 | 0.257 |
| BMI | 4.519 | 0.111 |
| T2DM duration | 1.388 | 0.720 |
| Smoke | 4.269 | 0.143 |
| Drink | 4.120 | 0.154 |
| Hypertension | 4.012 | 0.170 |
| Hyperlipidemia | 3.387 | 0.295 |
| Thyroid disease | 4.233 | 0.140 |
| Renal failure | 1.260 | 0.792 |
| HbA1c | 1.525 | 0.656 |
| eGFR | 21.432 | 0.049 |
| ALT | 1.188 | 0.841 |
| AST | 1.141 | 0.877 |
| TC | 1.449 | 0.690 |
| TG | 1.267 | 0.789 |
| HDL-C | 1.268 | 0.788 |
| LDL-C | 1.345 | 0.743 |
| FPG | 1.580 | 0.633 |
| 2hPPG | 1.755 | 0.570 |
| MPG | 2.478 | 0.404 |
| Operation duration | 1.176 | 0.851 |
| Nutritional support | 4.955 | 0.106 |
| Anesthetic mode | 2.287 | 0.437 |
| ASA grade > Ⅱ | 1.285 | 0.656 |
| Postoperative fasting time | 1.012 | 0.897 |
| PLOS | 2.230 | 0.448 |
| CRP | 1.236 | 0.809 |
| WBC | 1.350 | 0.741 |
| NEUT | 1.356 | 0.737 |
| Diuretic | 4.129 | 0.154 |
| β-blocker | 4.381 | 0.140 |
| Somatostatin | 3.856 | 0.262 |
| Quinolones | 3.963 | 0.234 |
| Previous prolonged insulin treatment | 1.833 | 0.539 |
| Postoperative insulin administration | 17.921 | 0.056 |

VIF: variance inflation factor; BW, body weight; BMI, body mass index; T2DM, type 2 diabetes mellitus; HbA1c, glycated haemoglobin; eGFR, estimated glomerular filtration rate; ALT, alanine transaminase; AST, aspartate transaminase; TC, total cholesterol; TG, triglyceride; HDL-C, high density lipoprotein cholesterol; LDL-C, low density lipoprotein cholesterol; FPG, fasting plasma glucose; 2hPPG, 2-hour postprandial plasma glucose; MPG, 3-day preoperative mean plasma glucose; ASA, American Society of Anesthesiologists; PLOS, postoperative length of stay; CRP, c reactive protein; WBC, white blood cell; NEUT, neutrophil

**TABLE S4** Univariate analysis of predictors for level 1 and level 2 after laparoscopic CRC resection in hospitalized patients with T2DM

| Variables | Level 1 hypoglycemia | | | | Level 2 hypoglycemia | | | |
| --- | --- | --- | --- | --- | --- | --- | --- | --- |
|  | Hypoglycemia  N = 98^a^ | Non-hypoglycemia  N = 600^a^ | OR (95%CI) | *p*^b^ | Hypoglycemia  N = 22^a^ | Non-hypoglycemia  N = 600^a^ | OR (95%CI) | *p*^b^ |
| **Demographic characteristics** | | | | | | | | |
| Age, y | 69.00 (64.00, 75.00) | 67.00 (59.00, 73.00) | 1.03 (1.01, 1.05) | 0.010 | 75.00 (67.00, 82.00) | 67.00 (59.00, 73.00) | 1.08 (1.04, 1.14) | < 0.001 |
| Sex, n, male | 45 (45.9%) | 336 (56.0%) | 0.67 (0.43, 1.02) | 0.064 | 6 (27.3%) | 336 (56.0%) | 0.29 (0.11, 0.76) | 0.012 |
| BW, kg | 57.50 (50.00, 65.00) | 60.00 (52.00, 67.75) | 0.98 (0.96, 1.00) | 0.068 | 56.50 (47.75, 62.00) | 60.00 (52.00, 67.75) | 0.97 (0.93, 1.01) | 0.122 |
| BMI, kg/m^2^ | 21.88 (19.70, 23.50) | 22.84 (20.58, 24.98) | 0.92 (0.87, 0.99) | 0.015 | 19.39 (18.06, 20.46) | 22.84 (20.58, 24.98) | 0.68 (0.58, 0.79) | < 0.001 |
| T2DM duration, y | 4.00 (1.00, 13.25) | 1.00 (1.00, 11.00) | 1.03 (1.01, 1.06) | 0.011 | 11.00 (1.38, 14.00) | 1.00 (1.00, 11.00) | 1.05 (1.00, 1.10) | 0.042 |
| Smoke, yes | 12 (12.2%) | 93 (15.5%) | 0.91 (0.49, 1.67) | 0.757 | 2 (9.1%) | 93 (15.5%) | 0.57 (0.13, 2.45) | 0.446 |
| Drink, yes | 4 (4.1%) | 23 (3.8%) | 1.35 (0.50, 3.64) | 0.554 | 1 (4.5%) | 23 (3.8%) | 1.18 (0.15, 9.13) | 0.872 |
| Comorbidities | 29 (29.6%) | 151 (25.2%) | 1.25 (0.78, 2.00) | 0.354 | 6 (27.3%) | 151 (25.2%) | 1.12 (0.43, 2.90) | 0.823 |
| Hypertension, yes | 1 (1.0%) | 16 (2.7%) | 0.38 (0.05, 2.87) | 0.346 | 0 | 16 (2.7%) | - | 0.502 |
| Hyperlipidemia, yes | 4 (4.1%) | 20 (3.3%) | 1.89 (0.74, 4.83) | 0.183 | 2 (9.1%) | 20 (3.3%) | 2.81 (0.62, 12.71) | 0.180 |
| Thyroid disease, yes | 9 (9.2%) | 18 (3.0%) | 4.09 (1.87, 8.95) | < 0.001 | 2 (9.1%) | 18 (3.0%) | 2.49 (0.55, 11.18) | 0.235 |
| Renal failure, yes | 4 (4.1%) | 16 (2.7%) | 1.53 (0.51, 4.75) | 0.436 | 1 (4.5%) | 8 (1.3%) | 3.52 (0.42, 29.47) | 0.215 |
| **Baseline laboratory tests** | | | | | | | | |
| HbA1c^c^, % | 7.05 (6.20, 8.33) | 7.03 (6.22, 8.22) | 1.07 (0.95, 1.22) | 0.258 | 7.00 (6.02, 7.88) | 7.03 (6.22, 8.22) | 1.04 (0.81, 1.35) | 0.742 |
| eGFR^c^, mL/(min·1.73m^2^) | 55.82 (40.77, 74.31) | 63.90 (49.35, 80.47) | 0.99 (0.98, 1.00) | < 0.001 | 45.37 (32.28, 59.73) | 63.90 (49.35, 80.47) | 1.73 (0.99, 3.01) | 0.054 |
| ALT^c^, U/L | 24.19 (14.30, 33.81) | 23.13 (14.56, 37.51) | 1.00 (0.99, 1.01) | 0.869 | 28.97 (19.66, 33.47) | 23.13 (14.56, 37.51) | 1.00 (0.98, 1.03) | 0.864 |
| AST^c^, U/L | 21.36 (16.44, 30.01) | 19.75 (15.23, 29.49) | 1.01 (0.99, 1.02) | 0.262 | 24.41 (18.29, 29.38) | 19.75 (15.23, 29.49) | 1.02 (0.99, 1.05) | 0.240 |
| TC^c^, mmol/L | 4.78 (4.13, 5.84) | 5.01 (3.84, 5.97) | 0.96 (0.83, 1.11) | 0.612 | 4.70 (3.62, 5.89) | 5.01 (3.84, 5.97) | 0.90 (0.67, 1.20) | 0.477 |
| TG^c^, mmol/L | 1.95 (1.32, 2.97) | 1.94 (1.29, 2.84) | 1.05 (0.95, 1.15) | 0.358 | 1.69 (1.22, 2.59) | 1.94 (1.29, 2.84) | 0.89 (0.67, 1.18) | 0.428 |
| HDL-C^c^, mmol/L | 1.01 (0.87, 1.20) | 1.05 (0.93, 1.27) | 0.82 (0.41, 1.63) | 0.567 | 1.02 (0.90, 1.16) | 1.05 (0.93, 1.27) | 1.24 (0.35, 4.43) | 0.737 |
| LDL-C^c^, mmol/L | 3.00 (2.41, 3.69) | 2.97 (2.30, 3.58) | 1.16 (0.93, 1.46) | 0.191 | 2.83 (2.07, 3.68) | 2.97 (2.30, 3.58) | 1.00 (0.64, 1.56) | 0.998 |
| FPG, mmol/L  (the day prior to operation) | 5.95 (5.10, 7.00) | 6.61 (5.49, 8.12) | 0.82 (0.73, 0.92) | < 0.001 | 5.19 (4.44, 5.77) | 6.61 (5.49, 8.12) | 0.60 (0.47, 0.78) | < 0.001 |
| 2hPPG, mmol/L  (the day prior to operation) | 7.80 (6.10, 9.42) | 7.88 (6.20, 10.23) | 0.99 (0.93, 1.06) | 0.825 | 9.19 (6.40, 10.00) | 7.88 (6.20, 10.23) | 1.04 (0.91, 1.19) | 0.582 |
| MPG, mmol/L | 7.52 (6.33, 9.61) | 7.79 (6.61, 9.11) | 1.03 (0.93, 1.14) | 0.800 | 8.28 (7.19, 10.51) | 7.79 (6.61, 9.11) |  |  |
| **Perioperative data** | | | | | | | | |
| Operation duration | 160.00 (145.00, 180.00) | 155.00 (140.00, 175.00) | 1.01 (1.00, 1.01) | 0.108 | 158 .00 (140.00, 180.00) | 155.00 (140.00, 175.00) | 1.00 (0.99, 1.02) | 0.939 |
| Nutritional support |  |  |  |  |  |  |  |  |
| EN | 19 (19.4%) | 76 (12.2%) | 1.73 (0.99, 3.01) | 0.054 | 2 (9.1%) | 76 (12.7%) |  |  |
| EN+PN | 19 (19.4%) | 83 (13.3%) | 0.92 (0.45, 1.86) | 0.807 | 2 (9.1%) | 83 (13.8%) | 0.92 (0.13, 6.66) | 0.931 |
| No | 40 (40.8%) | 308 (49.5%) | 0.52 (0.28, 0.95) | 0.033 | 14 (63.6%) | 308 (51.3%) | 1.73 (0.38, 7.76) | 0.476 |
| PN | 20 (20.4%) | 133 (21.4%) | 0.60 (0.30, 1.20) | 0.148 | 4 (18.2%) | 133 (22.2%) | 1.14 (0.20, 6.39) | 0.879 |
| Anesthetic mode |  |  |  |  |  |  |  |  |
| general anesthesia | 89 (90.8%) | 521 (83.8%) | 1.92 (0.94, 3.93) | 0.076 | 19 (86.4%) | 521 (83.8%) | - | - |
| general anesthesia and CSEA | 3 (3.1%) | 43 (6.9%) | 0.41 (0.12, 1.34) | 0.141 | 2 (9.1%) | 43 (6.9%) | 1.28 (0.29, 5.66) | 0.749 |
| general anesthesia and nerve block anesthesia | 6 (6.1%) | 36 (5.8%) | 0.98 (0.40, 2.38) | 0.957 | 1 (4.5%) | 36 (5.8%) | 0.76 (0.10, 5.85) | 0.794 |
| ASA grade > Ⅱ | 61 (62.2%) | 267 (42.9%) | 2.06 (1.33, 3.19) | 0.001 | 16 (72.7%) | 267 (42.9%) | 3.33 (1.28, 8.62) | 0.013 |
| Postoperative  fasting time, d | 5.00 (2.00, 8.00) | 3.00 (1.00, 7.00) | 1.05 (1.01, 1.09) | 0.026 | 7.50 (4.00, 11.00) | 3.00 (1.00, 7.00) | 1.11 (1.05, 1.16) | < 0.001 |
| PLOS, d | 6.00 (3.00, 10.00) | 5.00 (3.00, 8.00) | 1.05 (1.01, 1.09) | 0.014 | 4.50 (1.00, 9.00) | 5.00 (3.00, 8.00) | 1.01 (0.93, 1.09) | 0.810 |
| **Inflammatory parameters on POD 3** | | | | | | | | |
| CRP, mg/L | 109.90 (68.90, 143.38) | 97.50 (70.11, 126.00) | 1.00 (1.00,1.00) | 0.746 | 101.00 (84.33, 150.18) | 97.50 (70.11, 126.00) | 1.00 (1.00, 1.01) | 0.405 |
| WBC, 10^9^/L | 8.25 (5.75, 11.98) | 8.64 (6.56, 11.36) | 0.99 (0.94, 1.04) | 0.617 | 8.18 (6.78, 12.61) | 8.64 (6.56, 11.36) | 1.05 (0.97, 1.14) | 0.250 |
| NEUT, % | 83.03 (76.10, 86.85) | 80.76 (73.90, 85.53) | 1.02 (0.99, 1.04) | 0.160 | 85.61 (77.46, 90.02) | 80.76 (73.90, 85.53) | 1.07 (1.01, 1.13) | 0.025 |
| **Other perioperative medications** | | | | | | | | |
| Diuretic, yes | 6 (6.1%) | 17 (2.7%) | 2.64 (1.06, 6.54) | 0.036 | 1 (4.5%) | 17 (2.7%) | 1.40 (0.18, 10.84) | 0.749 |
| β-blocker, yes | 5 (5.1%) | 21 (3.4%) | 1.80 (0.71, 4.57) | 0.218 | 1 (4.5%) | 21 (3.4%) | 1.23 (0.16. 9.5) | 0.842 |
| Somatostatin, yes | 9 (9.2%) | 49 (7.9%) | 1.14 (0.54, 2.40) | 0.735 | 4 (18.2%) | 49 (7.9%) | 2.45 (0.80, 7.49) | 0.115 |
| Quinolones, yes | 2 (2.0%) | 11 (1.8%) | 1.69 (0.46, 6.17) | 0.427 | 1 (4.5%) | 11 (1.8%) | 2.51 (0.31, 20.08) | 0.386 |
| Previous prolonged insulin treatment^d^, yes | 30 (30.6%) | 134 (21.5%) | 1.53 (0.96, 2.46) | 0.075 | 17 (77.3%) | 134 (21.5%) | 11.82 (4.28, 32.64) | < 0.001 |
| Postoperative insulin administration, yes | 62 (63.3%) | 227 (36.5%) | 3.00 (1.93, 4.67) | < 0.001 | 11 (50.0%) | 227 (36.5%) | 1.64 (0.70, 3.85) | 0.253 |

^a^Cases (n, percentage/%) or median (interquartile range)

^b^Pearson’s chi-square test / Fisher’s exact test; Wilcoxon rank-sum test

^c^Testing on admission

^d^Preoperative continuation of pre-admission regimens

CRC, colorectal cancer; T2DM, type 2 diabetes mellitus; OR, odds ratio; CI, confidence interval; BW, body weight; BMI, body mass index; T2DM, type 2 diabetes mellitus; HbA1c, glycated haemoglobin; eGFR, estimated glomerular filtration rate; ALT, alanine transaminase; AST, aspartate transaminase; TC, total cholesterol; TG, triglyceride; HDL-C, high density lipoprotein cholesterol; LDL-C, low density lipoprotein cholesterol; FPG, fasting plasma glucose; 2hPPG, 2-hour postprandial plasma glucose; MPG, 3-day preoperative mean plasma glucose; EN, enteral nutrition; PN, parenteral nutrition; CSEA, combined spinal epidural anesthesia; ASA, American Society of Anesthesiologists; PLOS, postoperative length of stay; POD, postoperative day; CRP, c reactive protein; WBC, white blood cell; NEUT, neutrophil

**FIG. S1** Nomogram for predicting postoperative level 1 hypoglycemia in hospitalized patients with T2DM undergoing laparoscopic colorectal cancer resection

T2DM, type 2 diabetes mellitus; FPG, fasting plasma glucose

**FIG. S2** Nomogram for predicting postoperative level 2 hypoglycemia in hospitalized patients with T2DM undergoing laparoscopic colorectal cancer resection

T2DM, type 2 diabetes mellitus; FPG, fasting plasma glucose; NEUT, neutrophil
